# A Leaf Modeling and Multi-Scale Remeshing Method for Visual Computation via Hierarchical Parametric Vein and Margin Representation

**DOI:** 10.3389/fpls.2018.00783

**Published:** 2018-06-26

**Authors:** Weiliang Wen, Baojun Li, Bao-jun Li, Xinyu Guo

**Affiliations:** ^1^Beijing Research Center for Information Technology in Agriculture, Beijing, China; ^2^Beijing Key Lab of Digital Plant, National Engineering Research Center for Information Technology in Agriculture, Beijing, China; ^3^Faculty of Vehicle Engineering and Mechanics, School of Automotive Engineering, Dalian University of Technology, Dalian, China

**Keywords:** geometric modeling, multi-scale remeshing, plant leaf, vein, visual computation

## Abstract

This paper introduces a novel hierarchical structured representation for leaf modeling and proposes a corresponding multi-resolution remeshing method for large-scale visual computation. Leaf modeling is a very difficult and challenging problem due to the wide variations in the shape and structures among different species of plants. Firstly, we introduce a Hierarchical Parametric Veins and Margin (HPVM) representation approach, which describes the leaf biological structures and exact geometry via interpolation of parametric curves from the extracted vein features from non-manifold data. Secondly, a parametric surface model is constructed using HPVM with geometric and structured constraints. Finally, for a given size, we adapt a multi-step discrete point resampling strategy and a CDT-based (Constrained Delaunay Triangulation) meshing method to generate a mesh model. Our representation consists of three coupled data structures, a core hierarchical parametric data structure of veins and margin for the leaf skeleton, the corresponding parametric surface model, and a set of unstructured triangular meshes with user-specified density for the leaf membrane. Numerical experiments show that our method can obtain high quality meshes from the scanned non-manifold mesh data with well-preserved biological structures and geometry. This novel approach is suitable for effective leaf simulation, rendering, texture mapping, and simulation of light distribution in crop canopies.

## Introduction

Leaves are one of the most important structural and functional plant organs. Veins are composed of xylem and phloem cells embedded in parenchyma, sometimes sclerenchyma, and surrounded by bundle sheath cells (Sack and Scoffoni, 2013). These natural components form the optimized splitting structure of the leaf surface when aiming to create a model of a plant leaf. At present, the geometric modeling methods of plant leaves can be separated into the following categories: parametric modeling, L-System, 3D point cloud modeling, and image-based modeling. Surface reconstruction of plants is fundamental for functional-structural plant modeling (FSPM) (Vos et al., 2010; Henke et al., 2016). Model quality influences the accuracy and efficiency of further 3D model based visual computing including morphometric analysis of plants (Klein et al., 2017), 3D reconstruction of the plant canopy (Pound et al., 2014), estimating canopy gap fractions (Danson et al., 2007), simulating light distribution and analysis in plant canopies (Cieslak et al., 2008; Wiechers et al., 2011; Song et al., 2013), biochemical photosynthesis modeling and crop improvement (Hoon et al., 2016; Wu et al., 2016), modeling droplet pesticide residues on the leaf surface (Oqielat et al., 2011; Kempthorne et al., 2015), growth analysis and response of plants (Omasa et al., 2007; Jeong et al., 2013).

Leaf morphology affects modeling methods due to the different structures, shapes, and physiology. For instance, leaf veins are visible skeletons on the blade that act as conducting tissue and a support structure. Veins provide water and inorganic salt to leaves, and output photosynthesis products, while also supporting the blade, allowing it to extend in space. In addition, natural veins are the best mesh model of plant leaves. Leaf margins are the characterization of the blade edge. Therefore, veins and margins determine blade 3D characteristics, thus they are also important expression features in the process of parameterized modeling (Ogburn and Edwards, 2013; Sack and Scoffoni, 2013). However, current used geometric leaf models have omitted or used simulated venations. Ellatif (Ellatif, 2011) constructed leaf margins and veins using parametric modeling, and then optimized the value of the parameters to get the best representation of the considered leaf. Seo et al. (2014) generated different shapes of blades using L-System (Lindenmayer, 1968), then produced complete plants by combining different blades. 3D point cloud modeling (Paulus et al., 2014a) is one of the most widely used methods. It first constructs a leaf skeleton according to measured data, then deforms the skeleton to get a 3D model. Quan (Quan et al., 2006) reconstructed a single leaf model through point cloud data and images from different points of view. Tang et al. (2013) extracted margins from 2D images, then obtained a 3D leaf model by calculating Mass-Spring Model deformation in mesh. Interactive and simulation algorithms produce realistic leaves with veins (Hong et al., 2005; Runions et al., 2005; Alsweis et al., 2017), but there is a great difference between the simulated and the actual leaf venations. If the leaf mesh has only been subdivided in the skeleton (margin and veins) (Lu et al., 2009), the mesh could only be used for texture mapping, instead of the large scale visual computing for high precision. Auxiliary surface is another significant feature in leaf 3D modeling. Parameter surface modeling technology is the basis of the surface modeling system, its advantages lie in its precise control and convenience to draw. NURBS surface modeling (Hughes et al., 2005) is the core of parametric surface modeling. A NURBS (Non-Uniform Rational B-Spline) surface is the most general of the B-Spline methods. The particular interest here is the reconstruction of leaf surfaces from 3D scanning data under the constraint of palmate venation to form important components of virtual plants that are used in multi-scaled computational models for light distribution within plant canopies (Wiechers et al., 2011; Qian et al., 2014).

Simulating light distribution and accumulation in the plant canopy is an important application in FSPM. The process of simulation is time consuming, because a plant growing season can last several months and is subdivided into days, hours, and even into minutes. Geometric canopy models used in such simulations always incorporate a plant replication strategy to realize the marginal effect as this will greatly increase the computational complexity. However, dense geometric meshes of external (border) plants contribute only little to the simulation accuracy compared to sparse meshes. Therefore, a hierarchical geometric canopy model (in which the considered center plants are represented by a dense mesh, whereas for external plants a sparse mesh is chosen) is needed to realize an effective simulation and verification of light distribution (Wen et al., 2015). Current existing modeling methods of blade mesh are mostly of poor quality with a lot of characteristic geometric information lost. It is also difficult to add characteristic information back into the subsequent simulation process, thus a novel modeling procedure and analysis method based on feature preserving is needed.

Surface meshing is significant for numerical simulation and its related processing. In computer graphics, classical Delaunay triangulation (DT) is usually used to construct high quality mesh (Golias and Dutton, 1997). Some scholars used a local subdivision method to improve the quality of Delaunay triangular mesh (Ruppert, 1995). In addition, the most widely used meshing method is the Advancing Front Method (AFM) (George and Seveno, 1994). It is capable of generating new meshes independently by using the given geometric information. Although these two meshing methods differ in the input, they cannot guarantee that the mesh model will maintain the original geometric features. However, constrained Delaunay Triangulation (CDT) (Gudmundsson et al., 2004; Chen et al., 2012) can maintain these features to a certain extent. In the industrial field, offsetting features is usually adopted to preserve features with a simple shape. Another option to create a high quality mesh is simplifying the existing mesh, which transforms a given polygonal mesh into another mesh with fewer faces, edges, and vertices. There are many simplification methods, but the vertex clustering method (Tsuchie et al., 2014) and quadratic error measure (QEM) method (Garland and Heckbert, 1997) are most widely used. Hou et al. (2016) simplified field-grown maize and tobacco leaves using vertex removal and edge collapse methods, but the authors did not consider leaf venation features.

Specifically, an optimized target model should (i) focus on feature preserving and (ii) be hierarchical. Therefore, in the present study, morphological features of venation and leaf margins are considered. These features influence the normal distribution of leaf meshes. Photosynthesis simulation based on 3D light distribution models is seriously affected due to the inaccuracy of the geometric model, especially the normal of leaf meshes, thus leading to the accumulation of numerous errors. To improve the models, remeshing technology in computer graphics could be used to generate a hierarchical model.

This paper proposes a novel hierarchical structured representation for leaves with palmate venation and a corresponding multi-resolution CDT-based remeshing method for large-scale visual computation. Our numerical experiments show that this novel method can obtain high quality meshes from the scanned non-manifold mesh data while preserving biological structures and geometry, making the method suitable for effective leaf simulation, rendering, texture mapping, and simulation of light distribution in a crop canopy.

## Materials and methods

### Overview

**Figure 1:**
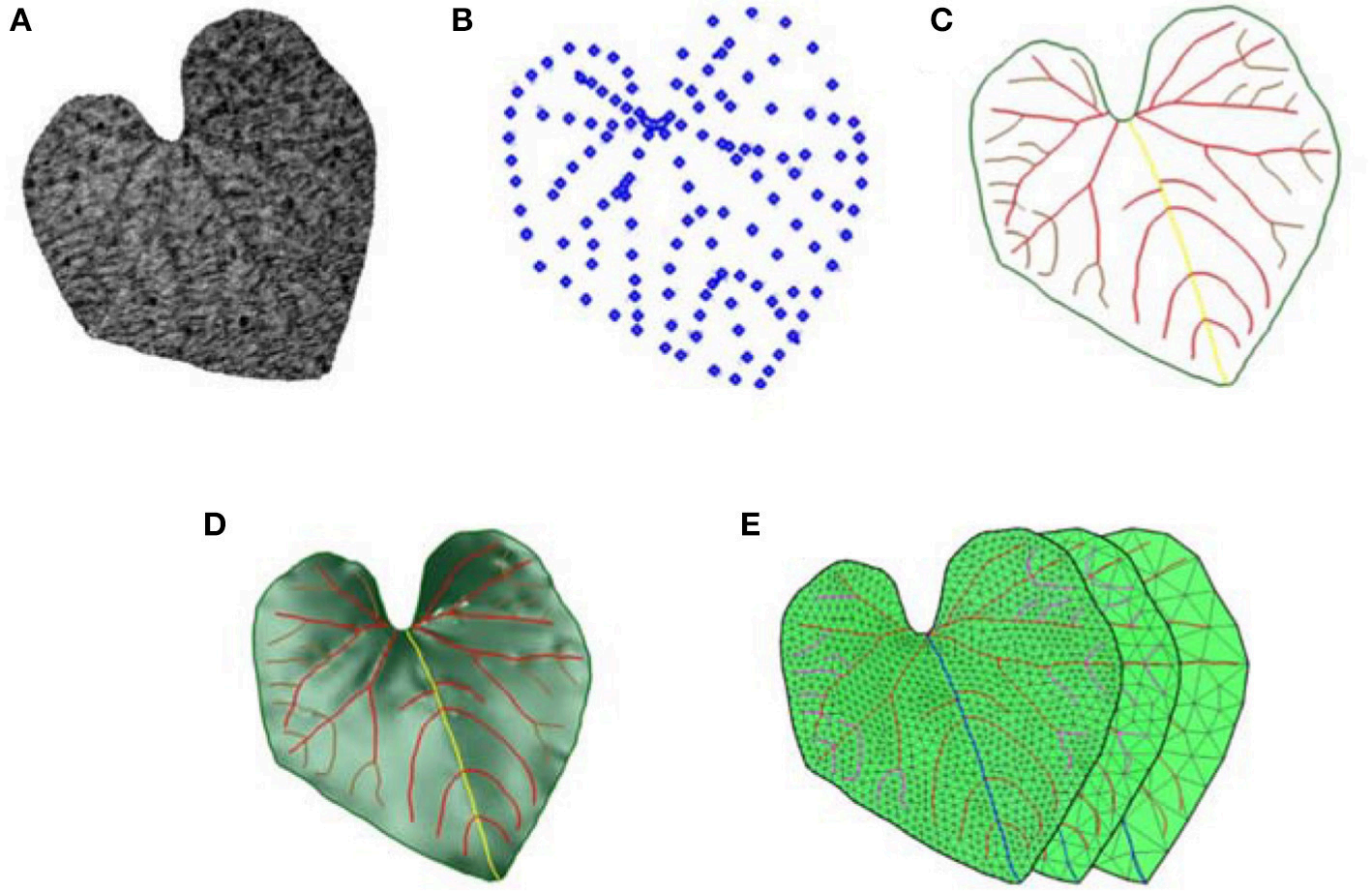
Data processing work flow of leaf 3D modeling via HPVM. **(A)** The input point cloud or original mesh model. **(B)** Feature points extracted from model A for HPVM construction. **(C)** HPVM model generation. **(D)** 3D parametric surface modeling via HPVM model. **(E)** HPVM-driven multi-scale meshing results using the models **(A,C)**.

Cucumber (“Green spirit”) and two types of grape (“Vitis amurensis” and “Cabernet sauvignon”) were grown in the greenhouse, and initially selected because the leaves have obvious venation. The handheld Artec Spider™ 3D scanner (Artec Group, Inc., Luxembourg) with a resolution of 0.1 mm was employed to capture 3D point clouds from selected leaves. The scanning process was conducted very slowly to avoid any displacement of the leaves, which would produce excessive noise. The processing of the point cloud registration and denoising were conducted using Artec Studio, supporting software for Artec scanners. Figure 1A is the visualization of the point cloud of a cucumber leaf with obvious venation feature points that could be selected in subsequent processes.

Our method consists of four main processing steps: HPVM construction, 3D parametric surface modeling, point resampling and mesh model generation (Figure 1).

When inputting a leaf scanning model, the first process was to generate the HPVM model by extracting its feature points in the first step. Those feature points were mainly located at veins and the margins (Figure 1B), thus the data volume shrunk by nearly 90% compared to the size of the point cloud during the extraction process. Feature points were then connected by B-spline curves according to their original order and code curves with their corresponding structure properties, with the HPVM model being the output. Due to the location of feature points, HPVM could be described as the leaf skeleton (Figure 1C). Next, an auxiliary surface constrained by HPVM curves was generated. HPVM curves and auxiliary surface composed the 3D leaf model (Figure 1D). The third step was to sample from the 3D surface model. Several parameters were introduced to control sampling distance and coordinate sampling points, such as element size, relaxing factor, and ignore factor. Resampling from HPVM curves was the most challenging step due to the complex connection within vein curves. The HPVM points were first sampled due to their critical role in mesh generation, and if those points were not sufficient for mesh generation, samples from the auxiliary surface were added as supplement points. Both HPVM sampling points and surface sampling points were integral in a constant scale.

In order to preserve leaf veins and the margins in the meshing step, a classical CDT method was adopted to generate a series of multi-scale meshes (Figure 1E). HPVM sampling points were connected by their original order as constrained polygons. The mesh generated by the CDT method was then optimized to get a high-quality mesh with the features preserved. The process and results are shown in Figure 1.

### Extracted HPVM model

**Figure 2:**
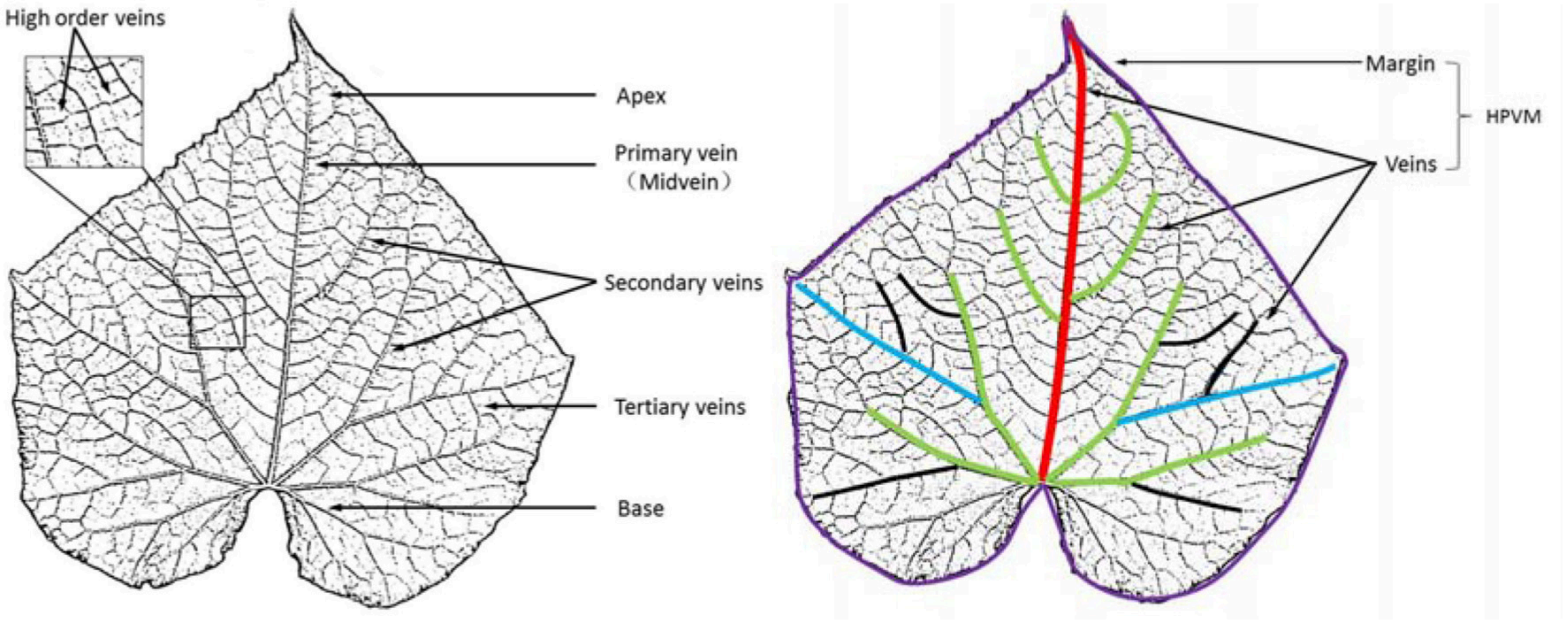
HPVM model extraction of a real leaf. The colored curves denote different level veins and the leaf margin.

The hierarchical parameterized veins and margin model was extracted to represent 3D geometric characteristics of blades from a real leaf (Figure 2). Leaf morphological features included both the HPVM model and the auxiliary surface.

#### Blade morphological feature structuring

**Figure 3:**
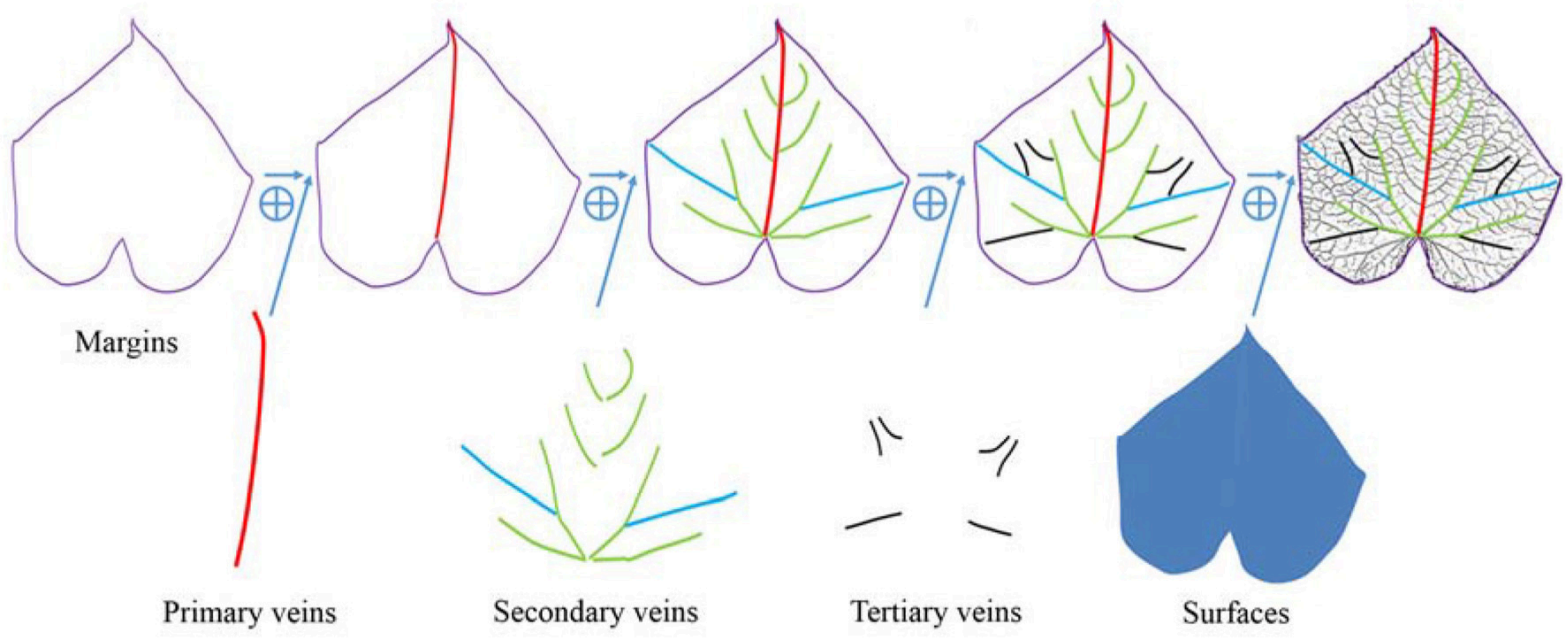
Morphological structural representation of a leaf. From left to right, the margin of the leaf boundary and the leaf normal, the primary veins for the left main direction, the secondary and tertiary veins for more details. These features were used to construct the HPVM model, generating the parametric surface.

As for the HPVM model, this primary vein is the largest gauge and generally runs from the base or near the base of the leaf to its apex. The start and end point of the primary vein are considered to be located at the margin. Major secondary veins (rib-forming veins) originate on the primary vein and run toward the margins. Interior secondary veins branch from major secondary veins and run toward the margins. Tertiary veins run from secondary veins. Leaf shape is also affected by dips near the margins and veins, so a ridge crest was introduced to express wrinkles. Thus, according to the dependence relationship described above, the feature hierarchy of a leaf from low to high can be identified as follows: margin, wrinkles, primary veins, secondary veins, tertiary veins and surface (see Figure 3).

#### Parametric expression of the leaf structural features

The HPVM model is composed of a series of curves. The B-spline was applied as the geometric basis to express the HPVM model. Thin leaf surfaces could be considered the geometric surface restrained by the HPVM model, while the NURBS surface was used to express the leaf surface in the process of the exact parameterization (Figure 3).

**Figure 4:**
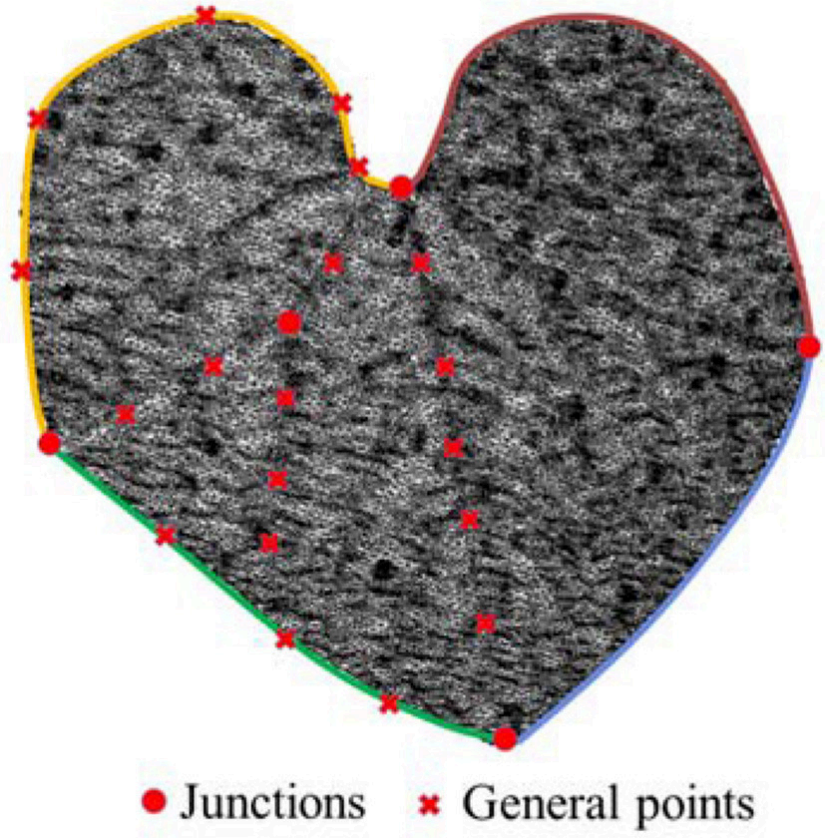
Feature points were extracted interactively by the user. Boundary points were used to generate the margin, and other points were used to generate the hierarchy veins. Junction points were marked for constraints in the surfacing and meshing step.

Contrary to the process of structuring, parameterization was processed from lower level features to higher level features. The first step in constructing the HPVM model was parameterization of the margin. In the modeling process, the margin was divided into several parts according to sharp points. Two sharp points were noted for the cucumber leaf: on the left and right sides besides the apex and base. Therefore, the leaf margin was divided into four parts (Figure 4). Each part was then interactively extracted using feature points from the scanned model. Leaf margin curves were obtained by fitting those feature points through a B-Spline.

The vein system provided the most important shape information of the leaf, and was the main component of the HPVM model. In the vein parameterization process, the start and end points determined the main direction of the veins; the ending points of the veins were mostly free points close to the margin. In the HPVM model, the beginning points of the veins were mostly considered a lower level feature. Points contacting two feature levels, which were the important feature points, could not be omitted in the structuring and parameterization process. The extraction process of vein feature points was similar to the extraction process of the margin. Additionally, structural feature points were extracted concurrently, thus the starting points followed by the ending points and bench points were extracted for every vein.

Wrinkles are common features of most margins. For relatively flat leaves, such as cucumber, we found that wrinkles could be expressed effectively with the constraints of margins and veins. It was not necessary to establish the wrinkle feature level. Therefore, a ridge crest line was introduced as a feature line, which enabled the model to express all leaf features. The process of the leaf parametric modeling method based on measured data is summarized in Figure 4.

### Auxiliary surface generation

A NURBS surface by closed margin curves was created and constrained by passing through the HPVM model. In the process of leaf surface parametric modeling, the HPVM model was used as guiding information. The main idea of the modeling process could be described as follows: a collection of parametric points was obtained from the HPVM model; these are constraint points in the NURBS surface. The parametric points were connected with a weight function, and the margin curve was used as the surface boundary. Then a leaf surface 3D model was obtained (Figure 4).

NURBS surface is defined as:

(1) $$p(u,w)=\frac{\sum_{i\;=\;0}^{m}\sum_{j\;=\;0}^{n}N_{i,k}(u)N_{j,l}(w)W_{ij}P_{ij}}{\sum_{i\;=\;0}^{m}\sum_{j\;=\;0}^{n}N_{i,k}(u)N_{j,l}(w)W_{ij}}$$

where *p*_*ij*_ is a control point, *W*_*ij*_ is a weight factor, *N*_*i, k*_(*u*) and *N*_*j*.*l*_(*w*) are basic functions of the B-Spline, and *m* and *n* are the orders.

The recursive basic function is defined as:

(2) $$\{\begin{array}{l} N_{i,0}(u)=\{\begin{array}{ll} 1, & u_{i}\leq u\leq u_{i+1}\\ 0, & else \end{array}\\ N_{i,k}(u)=\frac{u-u_{i}}{u_{i+k}-u_{i}}N_{i,k-1}(u)+\frac{u_{i+k+1}-u}{u_{i+k+1}-u_{i+1}}N_{i+1,k-1}(u),\;k\geq 1\\ \frac{0}{0}=0 \end{array}$$

where *k* is the order of the basis function, *u*_*i*_is a node, *i* = 0,1,…,*m*.

In the process of leaf surface parametric modeling, HPVM is used as guiding information. The modeling process followed a series of sampling of parametric points from the HPVM model, which were constraint points of a NURBS surface. These parametric points were connected with a weight function, taking the margin curve as a boundary. The final output was a 3D leaf auxiliary surface model.

### Resampling from a 3D leaf model

Triangular mesh remeshing is an important part of visual computing. The points were essential for mesh generation; therefore, high-quality, accurate points are required for mesh generation. Leaf multi-scale sampling points were obtained from different input parameters and were used to determine the sampling regulation such as the element size, relaxing factor, and ignore factor. In order to preserve leaf features, the sampling process was separated into two parts: HPVM sampling and auxiliary surface sampling.

#### HPVM model sampling

**Figure 5:**
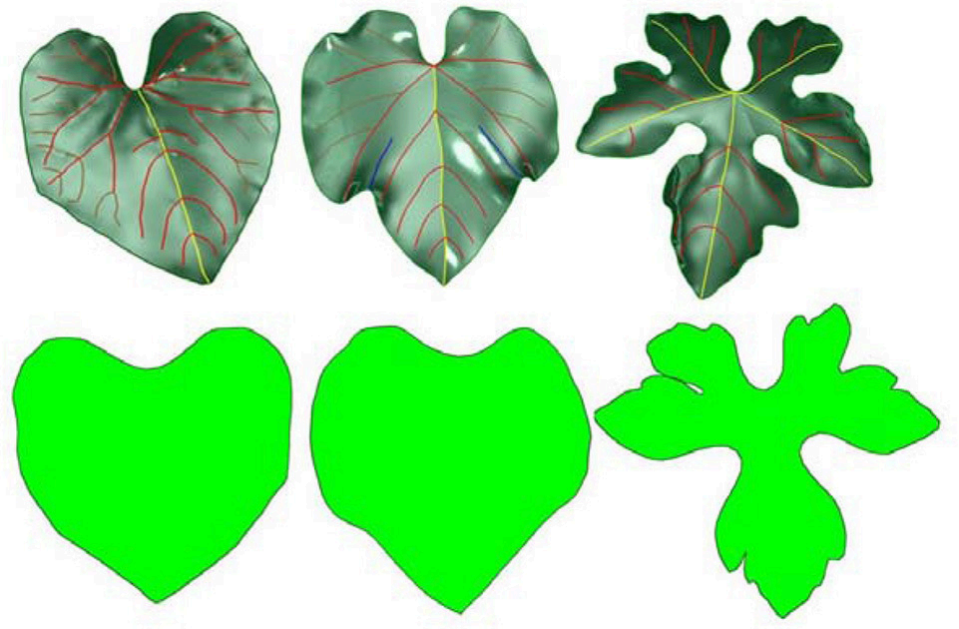
3D surface models of different leaves **(top)** and their corresponding 2D parametric domains **(bottom)**.

Points were sampled from the HPVM model. The *uv* coordinate system was used instead of the traditional *xyz* 3D coordinate system during the sampling process, thus the problem was transformed into the 2D space (Figure 5).

According to classification, points were sampled from each curve on the basis of the parameters, setting the starting points as reference. The most important parameter was the element size *l*, which controls the density of sampling points. When *l* is given, the points were sampled from the curve with the same arc length. A parameter defined as the ignore factor α was used to decide if a curve should be sampled or not. If the length of the curve was <α^*^l, it was not sampled.

**Figure 6:**
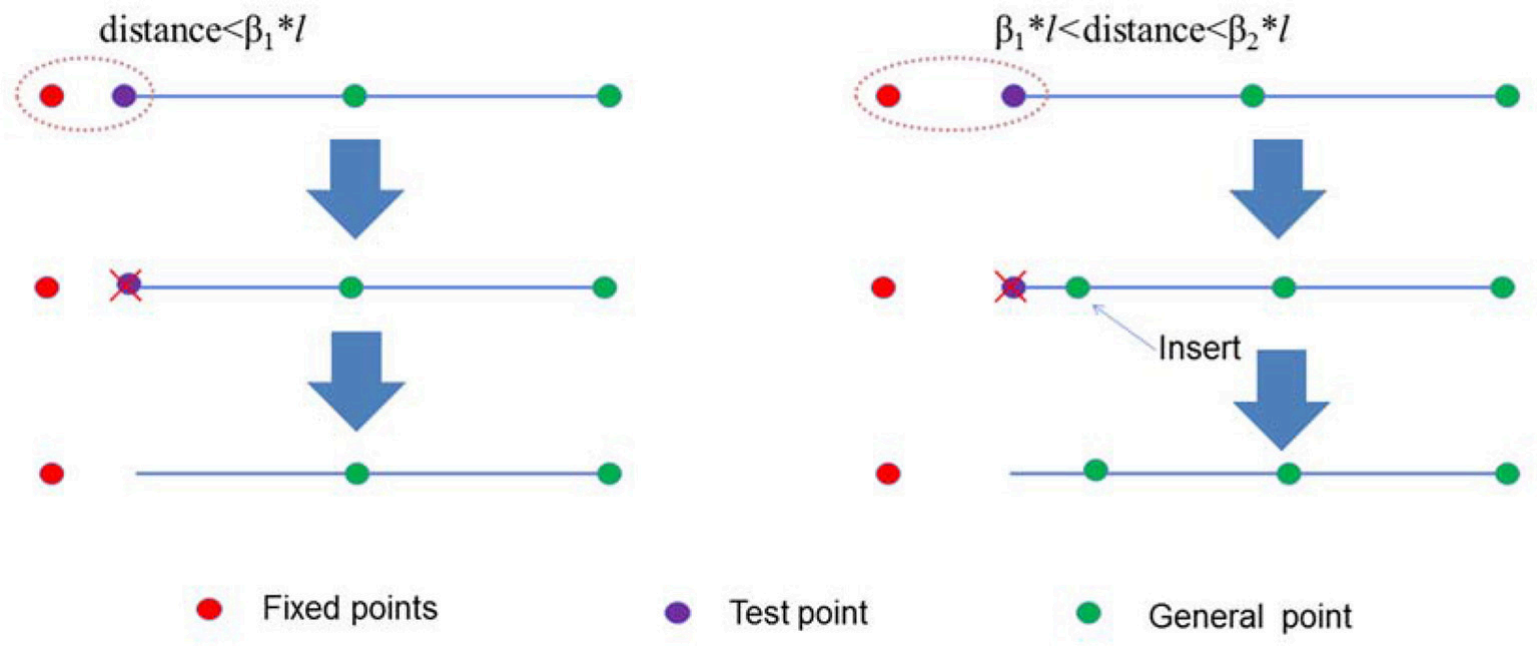
Illustration of a test point that should be deleted or inserted. If the distance between the test point and the fixed point was < β_1_*l, the test point was deleted. If the distance between the test point and the fixed point was more than β_1_*l but < β_2_*l, then a new point was inserted.

The sampling points between different curves might be close to each other, so two additional parameters named relaxing factors β_1_ and β_2_ were introduced. Relaxing factors guaranteed the quality of sampling points. Sampling points were individually relaxed from the lower level to the higher level. The points that were relaxed became fixed points. If the distance between test points and fixed points was <$\beta_{1}^{*}$l, the points were deleted. If the distance between test points and fixed points was more than $\beta_{1}^{*}$l but <$\beta_{2}^{*}$l, the test point was deleted before a new point was inserted at (β_1_+β_2_)/2^*^l (Figure 6 and Table 1).

**Table 1:** Algorithm for sampling process.

1: **procedure** SAMPLINGLEAF(*C, S*,β_1_, β_2_, *l*)
2: **for all** curves ***C**_i_* in *C* **do**
3: Set *R_*ij*_*← div(***C**_i_, l*)
4: end for
5: **for all** points *p_*i*_* in *R_*ij*_* **do**
6: Set P*_*i*_*←coordinate (*R_*ij*_, β*_1_, β_2_)
7: **end for**
8: Set *Q_*ij*_*←divAFM(S, R*_*ij*_*,*l*)
9: **return** (*P, Q*)
10: **end procedure**

#### Auxiliary surface sampling

Surface sampling was the most difficult process in the program due to the complex distribution of HPVM sampling points. In order to obtain a high-quality mesh, the surface sampling needed to be coordinated to surface sampling points and HPVM sampling points. The AFM method (George and Seveno, 1994) was adopted to generate surface sampling points with the HPVM sampling points as origin boundary.

**Figure 7:**
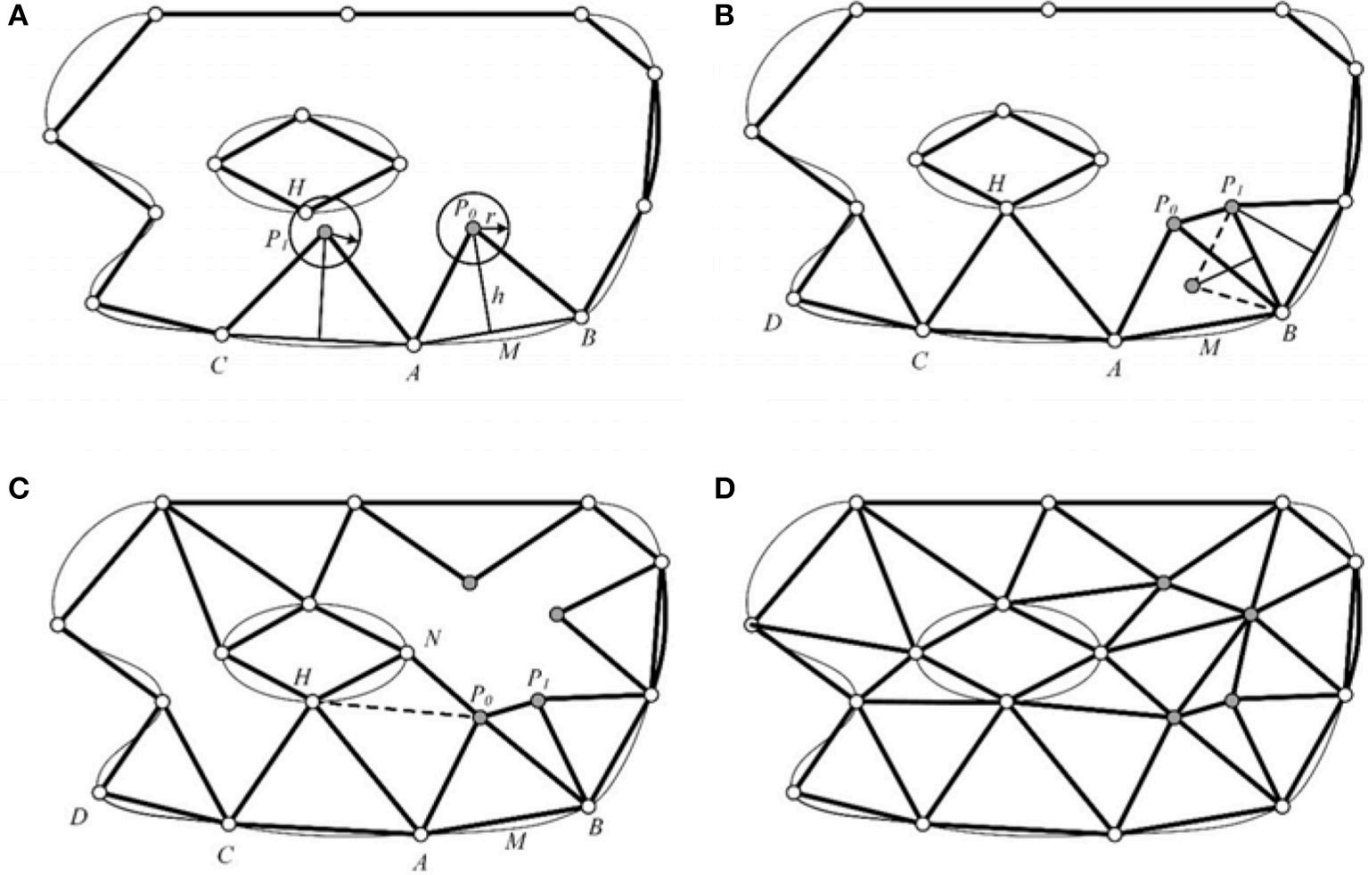
Illustration of AFM sampling workflow. **(A)** The input points and corresponding front set. A new node *P*_0_ is inserted from front *AB*. **(B)** The node *H* replaces the new inserted node *P*_1_ in Fig A, and another new node *P*_1_ is inserted. **(C)** Four new nodes are generated. **(D)** The final sampling and triangulation result.

The front set was built using the sampling points from the above section. As shown in Figure 7A, *AB* was selected as the active front, and a new point *P*_0_ was inserted along the perpendicular bisector of *AB* with a length of *h* $=\sqrt{3}*l/2$. Then, a search area was constructed with the given radius *r* and the center *P*_0_. If no other nodes were included in the search area, then the new node *P*_0_ was retained, and new active front *AP*_0_ and *BP*_0_, were simultaneously built. The front *AB* was deleted from the front set. The front *CA* forward was changed following the same methods to obtain a new node *P*_1_. Unfortunately, node *H* was included in the area with radius *r* and center *P*_1_. So, we choose *H* to replace the node *P*_1_, and build a triangle element *HAC*, then the new front *HA* and *CH* were built into the front set. Simultaneously, the *AC* front was deleted as shown in Figure 7B. Figure 7C illustrates the generation of four new nodes constructed using the above operation, to obtain a final triangulation mesh by AFM, shown as Figure 7D.

**Figure 8:**
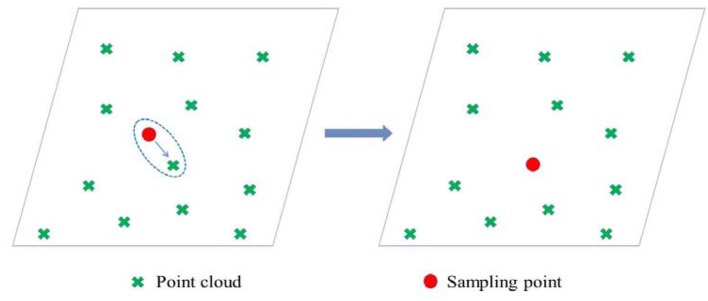
Position calibration of the sampling points. The sampling point (the red point in the figure on the left) was replaced by the closest one in the original model (the red point in the figure on the right).

Due to the locality of AFM, only sampling points were generated instead of concurrently generating a mesh in this paper. Accuracy is an important principle for remeshing, which means the reconstructed mesh must be as close as possible to the original model. Therefore, surface sampling points were calibrated with the origin point cloud. The leaf 3D surface model and the origin point cloud were added to the same coordinate system. When a new point was generated by the AFM method from the surface model, it was replaced by the closest point in the point cloud model. Thus, auxiliary surface sampling points could be regarded as sparse resampling of the point cloud model (see Figure 8).

### Mesh generation based on the CDT method

After the sampling point set was obtained, a discrete version of HPVM was built by connecting sampling points with the same topological structure as the HPVM model. In the meshing step, these lines were regarded as constraints. The mesh was then generated using the Constrained Delaunay Triangulation (CDT) method (Chew, 1987).

**Figure 9:**
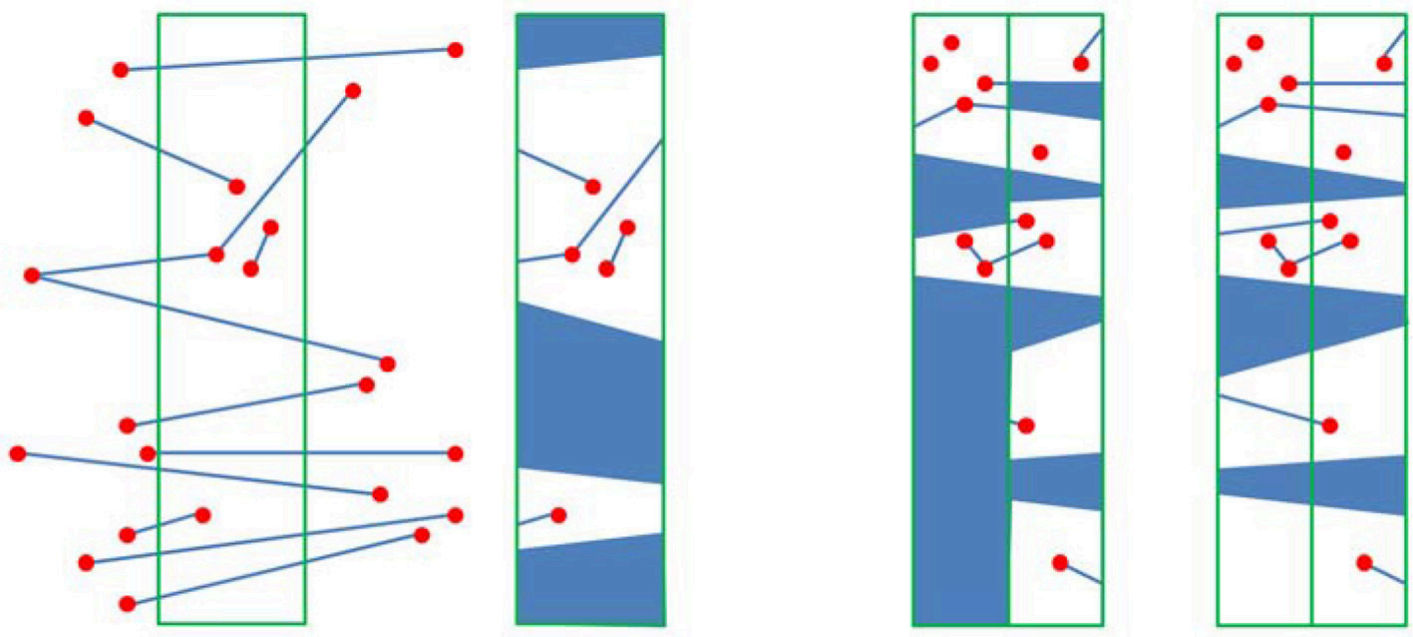
**Left**: The contents of a strip and the contents that were kept track of; **Right**: Merging the regions of two adjacent strips.

For a given set of *n* vertices with a set of non-crossing edges graph *G*, a CDT is the triangulation of the vertices with the following properties: (i) the pre-specified edges are included in the triangulation, and (ii) it is as close as possible to the Delaunay triangulation. It has been shown that the CDT can be built in optimal *O*(*n*log*n*) time using a divide-and-conquer technique. For simplicity of presentation, we assumed that the planar graph G was constrained with a given rectangle. The vertices of *G* were sorted by *u*-coordinate, and this information was used to divide the rectangle into vertical strips in such a way that there is exactly one vertex in each strip. Sampling could not proceed if a vertex was directly above another, so points were resampled in the *uv*-coordinate to avoid this problem. The CDT was calculated in each strip following the divide-and-conquer paradigm, and adjacent strips were pasted together in pairs to form new strips. The CDT was calculated for each newly formed strip until the CDT for the entire G-containing rectangle was built (Figure 9). This whole process used *O*(*n*log*n*) provided the CDT pasting operation was done efficiently.

## Results

### Analysis of geometric results

**Figure 10:**
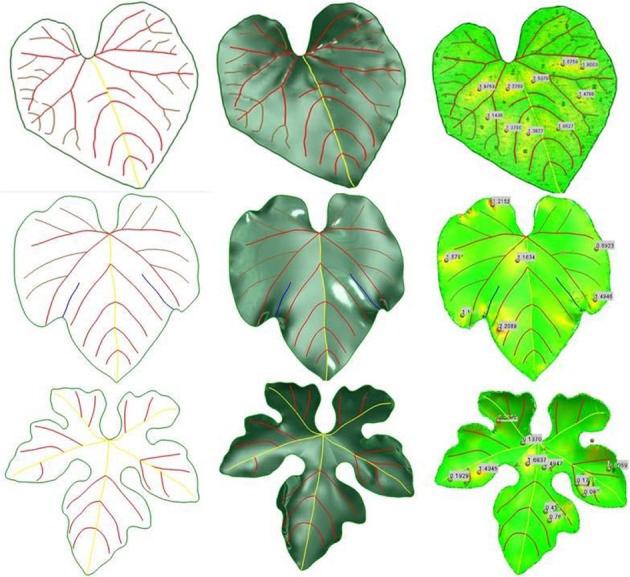
Three examples of 3D leaf modeling. From left to right, hierarchical parametric vein models, parametric surface models and error distributions respectively.

**Figure 11:**
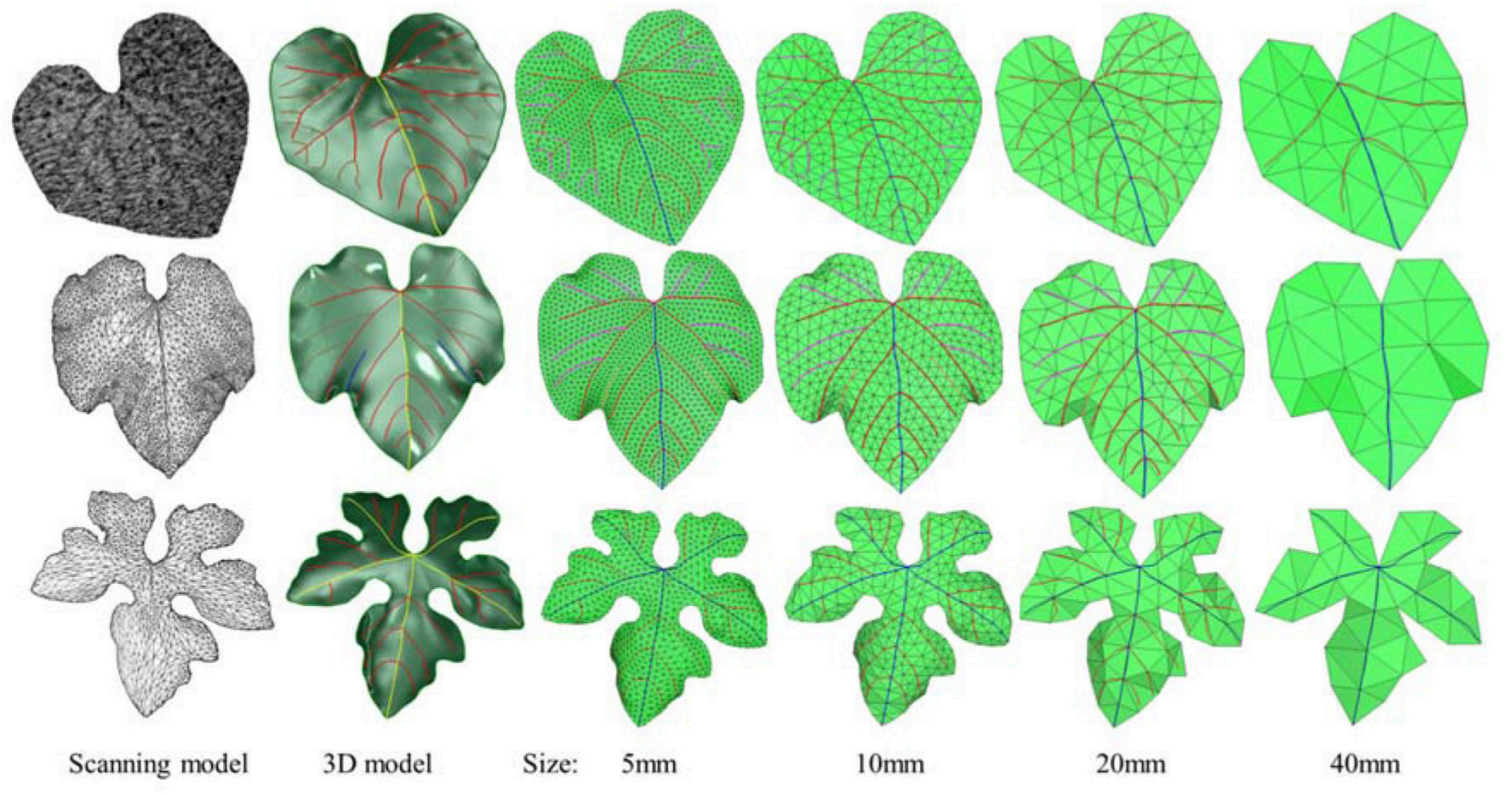
Three examples of 3D modeling and multi-scale meshing with our method. From left to right: the input original models, 3D parametric surface models with HPVM, HPVM-driven meshing results with size of 5, 10, 20, and 40 mm respectively.

We obtained multi-scale mesh models with preserved features using three 3D leaf models. Basic information is shown in Table 2, and the HPVM model, 3D leaf model, and error distribution compared with the scanning model are shown in Figure 10. The results indicate that when the elements are small, the elements mostly are regular triangles, and the HPVM model is well-preserved. When the size is more than 20 mm, tertiary veins and other short veins cannot be maintained and the loss of the boundary area becomes more pronounced. When the size continues to increase, more features are hard to preserve, and the quality of elements had to be sacrificed to better preserve features. The multi-scale mesh models are shown in Figure 11 and the error analysis is shown in Table 3.

**Table 2:** Statistics of three kinds of leaves.

**No**.	**Mid-vein length (mm)**	**Vertex number of scanning model**	**Number of feature points**	**Leaf area (mm^2^)**	**Maximum error (mm)**
1	142.84	38851	343	22689.35	2.52
2	158.75	4966	321	26147.24	3.10
3	128.12	4335	432	14937.29	2.15

**Table 3:** Statistics and analysis of mesh results.

**Object**	**Strains (size) mm**	**Element number**	**Percentage of pass [%]**	**Area error [%]**	**Maximum distance error (mm)**
1	5	2104	99.43	0.15	2.0343
	10	429	94.41	0.73	2.0711
	20	162	93.83	1.87	4.0517
	40	48	93.75	3.04	4.7725
2	5	1236	98.83	0.17	2.3125
	10	379	97.75	2.09	3.2997
	20	10	93.90	2.73	5.0908
	40	27	91.43	4.15	6.0246
3	5	1423	98.45	0.11	1.7491
	10	428	90.89	1.51	1.8121
	20	102	88.24	3.17	3.5779
	40	45	86.67	7.60	5.5555

Through the statistics and analysis of the mesh we found that when the element is small, the mesh is of high quality. With the number of element increasing, as a sacrifice to preserve HPVM features, mesh quality is decreasing. However, compared with the previous meshing method, which only sampled from skeleton, our method obtains a better mesh quality.

**Figure 12:**
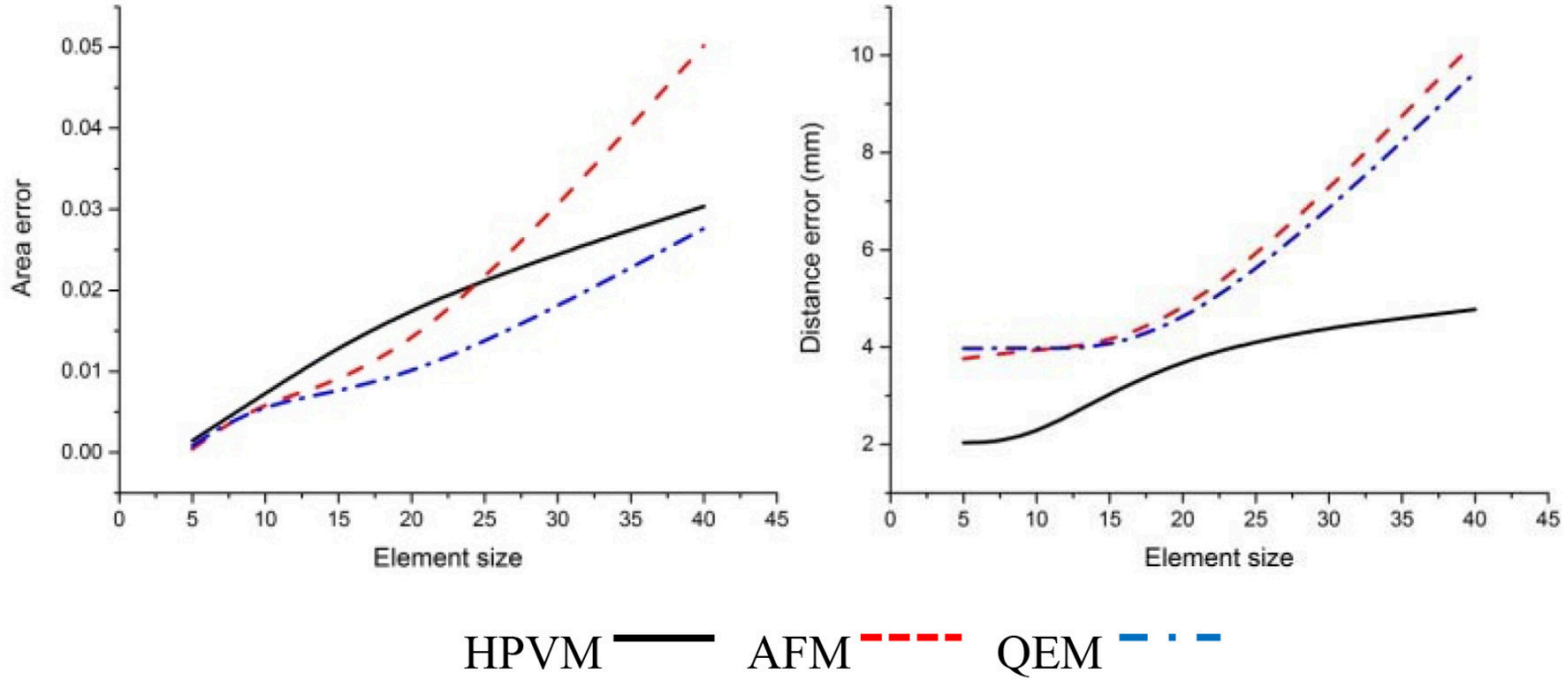
Comparisons of three kinds of meshing methods for the *cucumber* leaf. Numerical results show that HPVM method minimizes the distance error and holds the area error very well.

**Figure 13:**
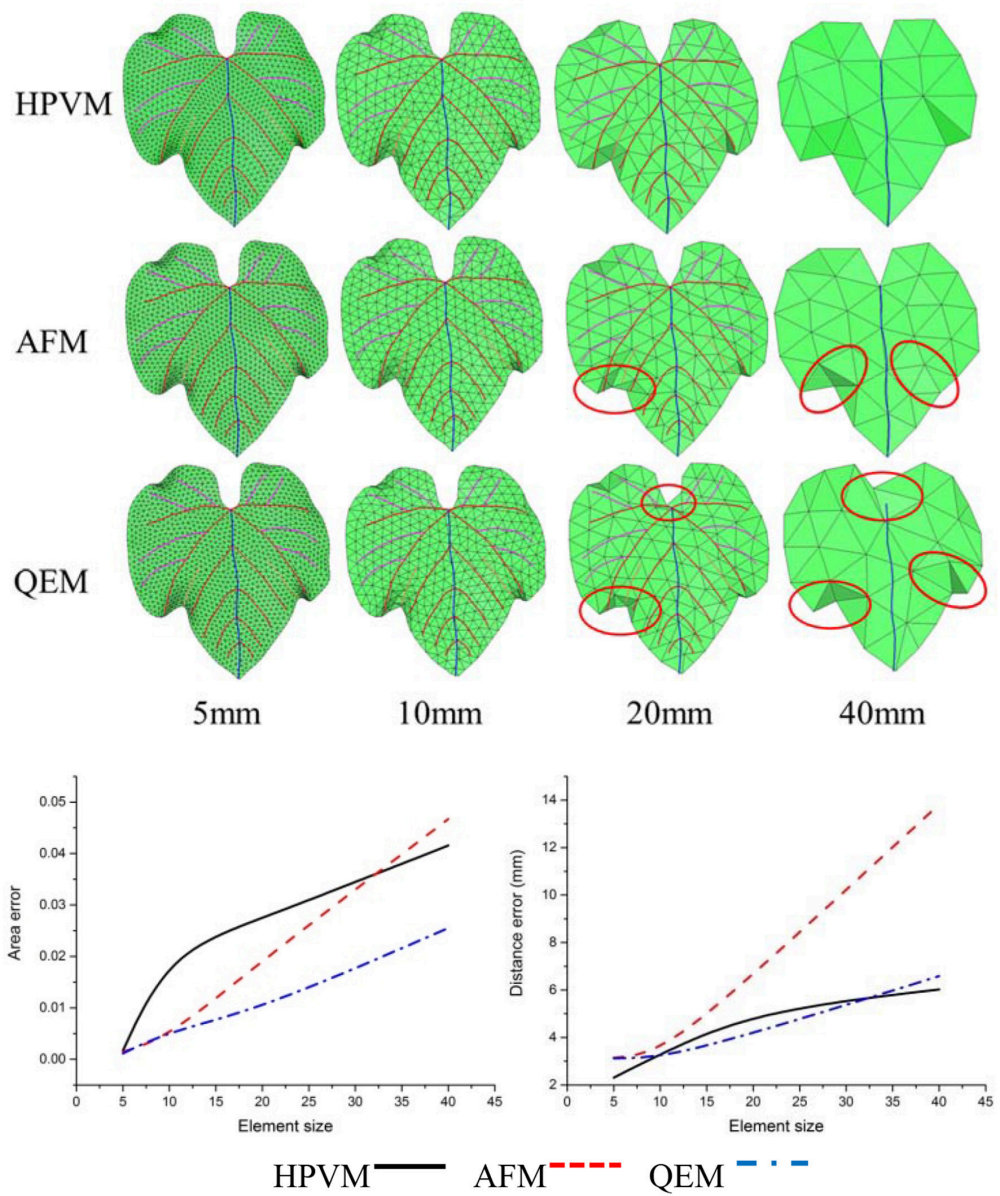
Comparisons of three kinds of meshing methods for “Vitis amurensis” leaf with different size respectively. Results show that with the HPVM method, improved morphological structures and details are obtained. Vein and margin features in circled areas are not well preserved using AFM and QEM methods especially when the size parameter becomes larger.

**Figure 14:**
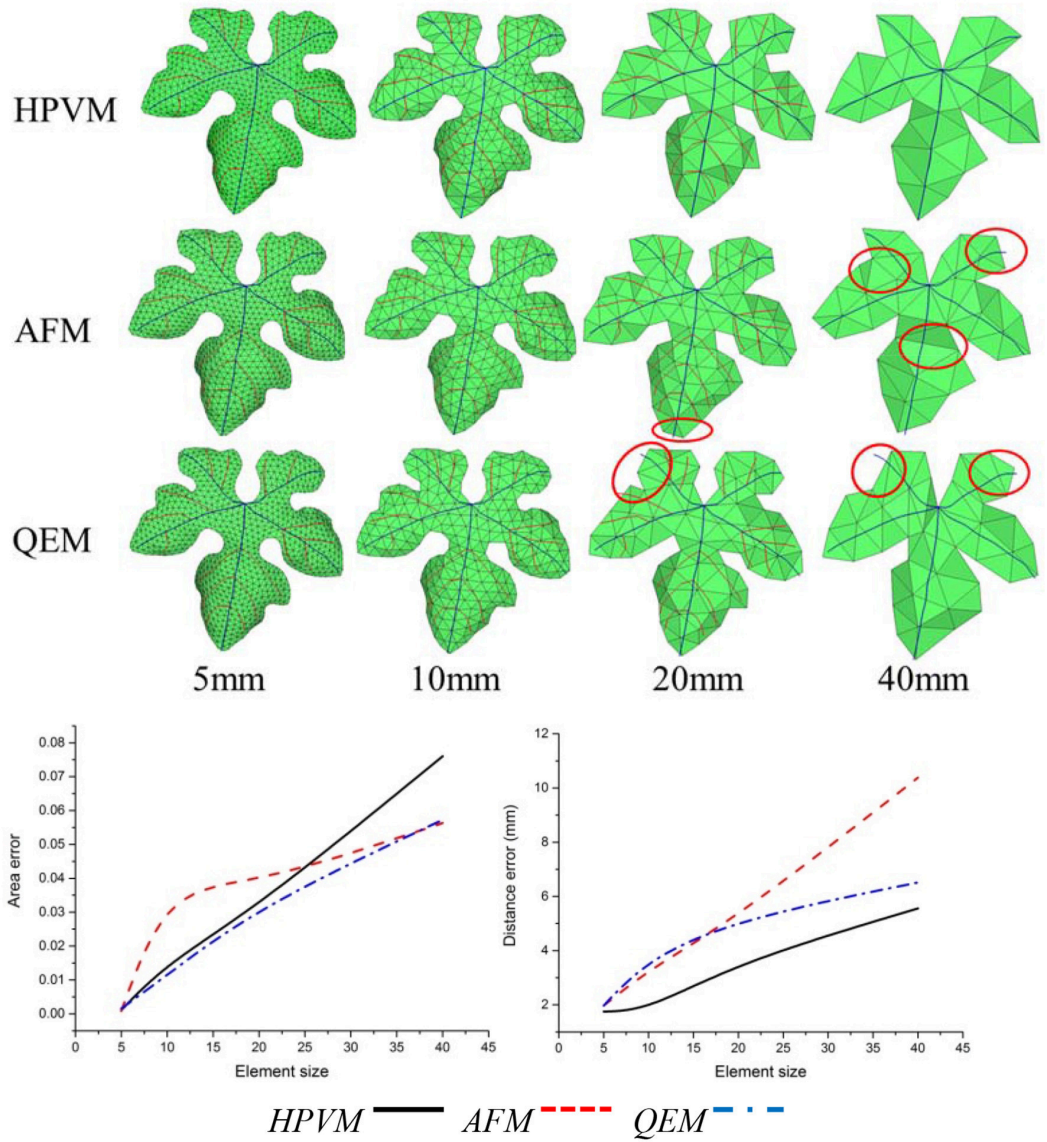
Comparisons of three kinds of meshing methods for “Cabernet sauvignon” leaf with different size respectively. Results show that with the HPVM method, improved morphological structures and details are obtained. Vein and margin features in circled areas are not well-preserved using AFM and QEM methods especially when the size parameter becomes larger.

In addition, in order to verify the effectiveness of the method, we adopted the AFM meshing method which is most widely used currently for the meshing surface, and the second algorithm which is the QEM method to simplify the original mesh model. Then we compared and analyzed three kinds of multi-scale meshes (see Figures 12–14 and Table 4).

**Table 4:** Comparison of three meshing methods.

**Objection method**	**Feature preservation**	**Element quality**	**Minimum area error**	**Minimum distance error**
HPVM	⋆⋆⋆	⋆⋆☆	⋆⋆☆	⋆⋆⋆
AFM	☆	⋆⋆⋆	⋆⋆	⋆⋆
QEM	☆	⋆⋆	⋆⋆⋆	⋆⋆

By comparing three kinds of meshing methods, we find that at the same scale, three methods exhibited the generally the same element number. With respect to morphology feature preservation, our method is more advantageous than the other methods. Furthermore, at the same scale, the mesh generated from HPVM-based method is close to the original mesh model. Namely, the HPVM meshing method is able to achieve the best approximation accuracy with the least number of elements. This fully shows the efficiency of the HPVM-based method.

### Simulating light distribution analysis

**Figure 15:**
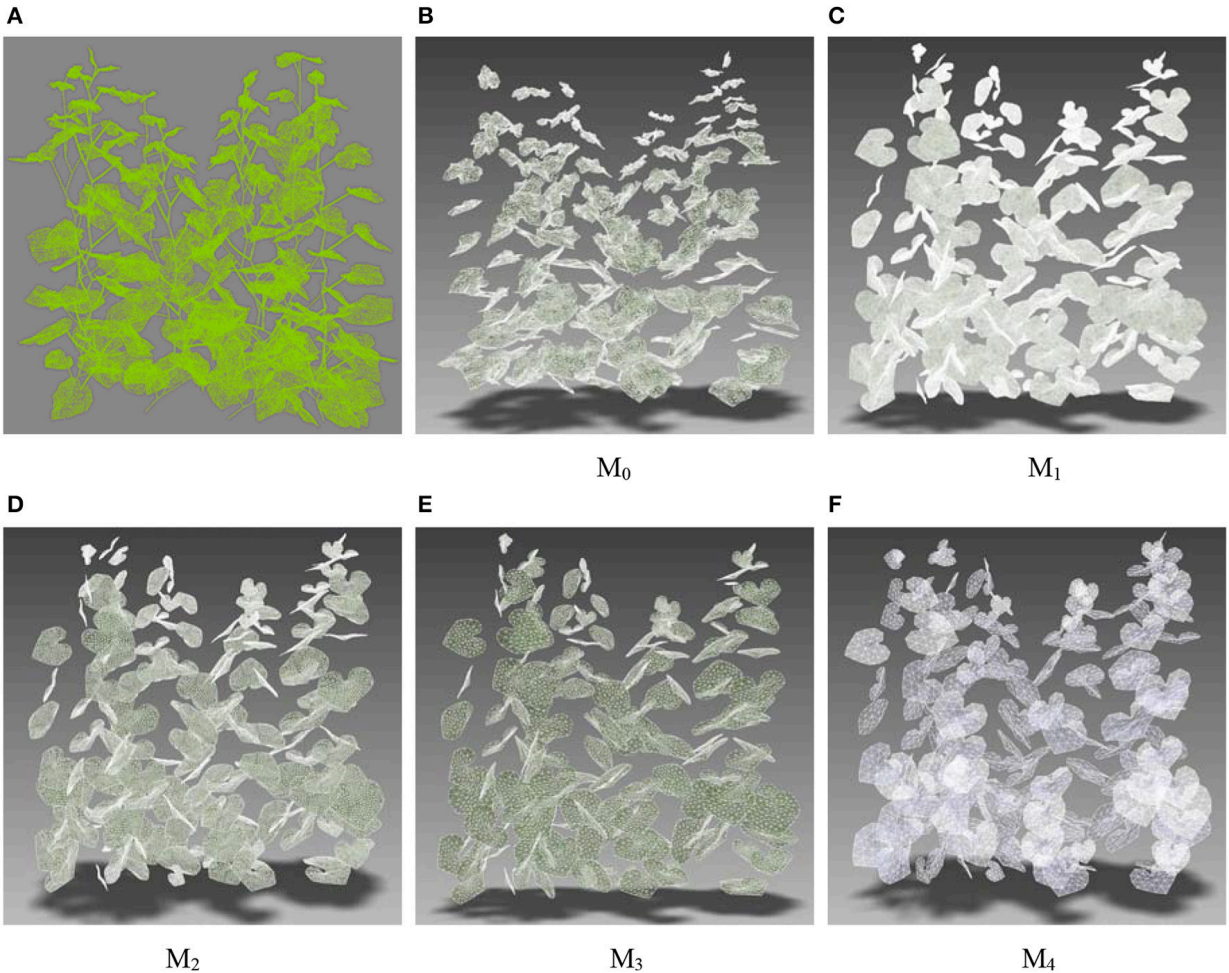
Multi-scale cucumber canopy models. **(A)** Original mesh with stalks; **(B)** Original mesh M_0_ without stalks; **(C–F)** Optimized and simplified meshes using HPVM method on M_0_, by setting different size parameters, abbreviated as M_1_, M_2_, M_3_, and M_4_ respectively.

An important application of this approach is to improve the computational efficiency of simulating light distribution in crop canopies. Due to the complexity of the crop canopy structure, more surface elements are needed to characterize the details of the canopy structure and plant organs in the canopy, which is essential to obtain a higher accuracy of simulating the light distribution. A geometric model of a cucumber canopy constructed by Qian (Qian et al., 2014) was used to illustrate the improved efficiency by using our method. The initial canopy model (M_0_) contains eight cucumber plants (2 rows × 4 plants in the rows). The spacing within and between rows was 40 cm. Stalk meshes were ignored for they contribute little to the simulation of light distribution while increasing the computational complexity. Geometric models of the leaves in M_0_ were acquired by 3D scanning and direct mesh simplification. The obtained meshes were messy and of low quality. By using the HPVM method on M_0_, we obtained four multi-scale canopy models M_1_, M_2_, M_3_, and M_4_ by setting the size parameter 5, 10, 20, and 40 mm respectively (Figure 15). As mentioned above, these groups of meshes were much more regular than the initial mesh and the quantities were controllable. As the input geometric model of the light simulation distribution algorithm used by Qian (Qian et al., 2014), 1,440 diffuse light sources uniformly distributed on a hemisphere were used to calculate the diffuse light distribution, which is a time-consuming process. The five groups of canopies have five different simulating efficiencies (Table 5). Results show that mesh simplification using the HPVM method can greatly improve the computational efficiency.

**Table 5:** Simulating efficiency of the different scale input canopy models.

**Model**	**Size parameter (mm)**	**Element quantity of the canopy**	**Element quantity per leaf**	**Time cost of simulation(s)**
M_0_		82893	499	74.162
M_1_	5	349265	2104	948.87
M_2_	10	71215	429	80.777
M_3_	20	26395	159	20.358
M_4_	40	7969	48	3.837

## Discussion

In this paper, a multi-scale modeling and remeshing method for the visual computation of palmate leaves, with examples from cucumber and grape, was described on the basis of 3D point cloud data. Compared with long, narrow cereal plant leaves (Paulus et al., 2014b; Wen et al., 2015), there are obvious multi-level venations on cucumber leaves and more margin features on grape leaves. To reflect the difference of margin features of different cultivars, two varieties of grape leaves representing diverse margin morphology were selected. A hierarchical parametric vein and margin representation approach was introduced, which provides both hierarchical geometric constraints for surface reconstruction and the subsequent multi-scale remeshing of leaf mesh. The meshing operation approach benefits from two significant characteristics: it preserves features and is multi-scale. These characteristics correspond to accuracy and efficiency, respectively, in subsequent visual computing applications.

Leaf veins and margins are important components of plant leaves, and their morphological structure reflects the ability of a plant to adapt to the environment. Veins contain transport nutrients and water within plants, and provide a structural support for leaves. Therefore, it is very important to preserve vein and margin features during the leaf mesh optimization process. Using conventional remeshing methods, such as QEM (Garland and Heckbert, 1997) and AFM (George and Seveno, 1994), directly may cause the loss of vein and margin characteristics in the resulting mesh (Figures 12–14). The optimized leaf mesh using the HPVM method has a high approximation to the vein and margin of the original mesh. The vertex removal and edge collapse methods performed on maize and tobacco leaves (Hou et al., 2016) mainly retained the curvature feature of the blade surface. The proposed HPVM method could also preserve the contours, mesh normal, and detail features of the leaf mesh. The remeshing method that preserves features is especially important for substantial simplification of leaves in a canopy. Our method provides high quality meshes to ensure the accuracy of the next step in visual computation such as biomechanical simulation (Von et al., 2015), canopy light distribution calculation (Mao et al., 2016), and pesticide residue simulation (Dorr et al., 2014).

With the development of the parameterized modeling theory and technology, a digital plant in a virtual simulation modeling realistic and controllable situations has further requirements. Therefore, introducing new geometric modeling methods from the computer graphics field and researching new modeling methods is suitable to be used for plant leaves. The main idea of the plant leaves model based on parameterized modeling (Wang et al., 2013; Zhang et al., 2017) is to extract parameters (morphological feature parameters) that could express blade geometric features. These morphological feature parameters express a 3D geometric blade model by polygons, Bezier, or NURBS curves and surfaces, which means the morphological feature parameters of the blade are determined by the control points or the weight factor of the NURBS surfaces. Users can realize different forms of 3D leaf models by modifying morphological feature parameters.

Crop canopies are composed of a large number of leaves. In the present study, the computation efficiency is directly related to the number of triangular facets used to describe the leaf surface. However, the reduction of the number of leaf mesh facets leads to the loss of detailed information, which causes the decline of calculation accuracy. Therefore, under the premise of ensuring calculation accuracy and maintaining as much of the leaf shape information as possible, it is important to improve the computational efficiency by reducing mesh number in FSPM research. Thus, leaf mesh optimization is important. In this paper, mesh optimization of multi-scale plant leaves was achieved by controlling the scale parameters. With less computational time cost, simulating light distribution in plant canopies would become a more practical tool in the research of analyzing light interception differences of different plant cultivars or planting densities. Compared with the vertex removal and edge collapse methods (Hou et al., 2016), the optimized mesh model is consistent. The difference between area or side length of the mesh facet is small, which is important for down-stream visualization.

The multi-scale vein extraction operation mainly depends on the manual completion described in this paper. The veins needs to be selected from each leaf point cloud interactively using our developed software, which is not automatic and limits the applicability of the method. Our future work aims to realize a point cloud based (Huang et al., 2013) leaf feature extraction algorithm to combine with the HPVM method to further achieve a more automatic feature preserving leaf mesh optimization program.

## Conclusions

This paper proposes a novel hierarchical structured representation for leaves and a corresponding multi-resolution remeshing method for large-scale visual computation. We introduce a Hierarchical Parametric Veins and Margin (HPVM) representation approach, which describes the leaf biological structures and exact geometry via interpolation of parametric curves from the extracted vein features. A parametric surface leaf model is constructed using the HPVM. For a given size, we adapt a multi-step discrete point resampling strategy and CDT-based meshing method. Our representation consists of three coupled data structures, a core hierarchical data structure of veins and margins (HPVM) for the leaf skeleton, the corresponding parametric surface model and a set of unstructured triangular meshes with user-specified density for the leaf membrane. Numerical experiments show that our method can obtain high quality meshes from the scanned non-manifold mesh data with well-preserved biological structures and geometry. This method provides a suitable solution to improve the computational efficiency, via reducing the number of leaf facets while preserving the morphometrics of plant leaves, in the 3D visualization model based functional structural plant analysis. It will be used to simulate and analyse the detailed light distribution differences in plant canopies caused by diverse leaf shape characteristics among different cultivars in future studies.

## Author contributions

All authors listed have made a substantial, direct and intellectual contribution to the work, and approved it for publication.

### Conflict of interest statement

The authors declare that the research was conducted in the absence of any commercial or financial relationships that could be construed as a potential conflict of interest.
